# A primitive actinopterygian braincase from the Tournaisian of Nova Scotia

**DOI:** 10.1098/rsos.171727

**Published:** 2018-05-16

**Authors:** Conrad D. Wilson, Jason D. Pardo, Jason S. Anderson

**Affiliations:** 1 Department of Biological Sciences, University of Calgary, Calgary, Alberta T2N 1N4, Canada; 2 McCaig Institute for Bone and Joint Health, University of Calgary, Calgary, Alberta T2N 1N4, Canada; 3 Department of Comparative Biology and Experimental Medicine, University of Calgary, Calgary, Alberta T2N 4N1, Canada

**Keywords:** braincase, Actinopterygii, Romer's Gap, Tournaisian

## Abstract

The vertebrate fossil record of the earliest Carboniferous is notoriously poorly sampled, obscuring a critical interval in vertebrate evolution and diversity. Recent studies of diversity across the Devonian–Carboniferous boundary have proposed a vertebrate mass extinction at the end-Devonian, and recent phylogenies suggest that the origin of the actinopterygian crown may have occurred in the earliest Carboniferous, as part of a broader recovery fauna. However, the data necessary to test this are limited. Here, we describe a partial actinopterygian skull, including diagnostic elements of the posterior braincase, from the Tournaisian Horton Bluff Formation of Blue Beach, Nova Scotia. The braincase surprisingly shows a confluence of characters common in Devonian taxa but absent in Mississippian forms, such as an open spiracular groove; lateral dorsal aortae that pass through open broadly separated, parallel grooves in the ventral otoccipital region, posterior to the articulation of the first infrapharyngobranchial and an intertemporal–supratemporal complex. Phylogenetic analysis places it deep within the actinopterygian stem, among Devonian moythomasiids and mimiids, suggesting more phylogenetically inclusive survivorship of stem group actinopterygians across the end-Devonian mass extinction. With a high lineage survivorship in tetrapods and lungfish across the Devonian–Carboniferous boundary and high vertebrate diversity at Tournaisian localities, this hints at a more gradual turnover between Devonian and Carboniferous vertebrate faunas.

## 1. Introduction

The Palaeozoic is marked by several biodiversity crises and major climatic events, including mass extinctions and longer-term transitions between climate states [1]. The Late Devonian is marked by two mass extinctions, one at the Frasnian–Famennian boundary (the Kellwasser event) and a second at the end-Famennian (the Hangenberg event) [2]. The faunal turnover occurring across the Hangenberg event includes the extinction of 31% of marine genera [2]. Together, the Kellwasser and Hangenberg events form a biodiversity depletion originally considered as one of the ‘Big Five’ mass extinctions [3].

Understanding in better detail how the vertebrate fauna responded to these mass extinction events has been hampered by the poor vertebrate fossil record immediately following the Devonian–Carboniferous boundary and extending into the Early Viséan. This gap in the fossil record, termed ‘Romer's Gap’ [4], has obscured critical events in vertebrate evolution, including the tetrapod fin–limb transition and the aftermath of the end-Devonian biodiversity crisis. Although some workers have suggested that Romer's Gap may represent either an interval of low atmospheric oxygen content [5] or a post-extinction recovery trough [6], recent intensive work in the earliest Carboniferous tetrapod fossil record has shown that tetrapod faunas, where preserved, are highly diverse and contain representatives of taxa typical of the Devonian and the later Carboniferous [7–9]. This trend is replicated in lungfish: new Tournaisian faunas reveal previously unrecognized lungfish diversity [10], in line with recent phylogenetic analyses that also indicate high lineage survivorship across the Devonian–Carboniferous boundary for lungfishes [11,12]. These new discoveries from the Tournaisian indicate that the Devonian–Carboniferous sarcopterygian transition may have been gradual, and that certain diversity trends in the Early Carboniferous may be an artefact of sampling bias.

Despite this survivorship, it is clear that a major vertebrate faunal transition occurs across the Devonian–Carboniferous boundary [13]. Actinopterygian diversity increases dramatically in the earliest Carboniferous, with a corresponding increase in morphological disparity and ecological role [13,14]. However, much of the earliest Carboniferous actinopterygian diversity has been assigned to non-neopterygian actinopterygian wastebasket taxa (e.g. [13,15]), of which only a minority have been treated in phylogenetic analyses to date. This lack of phylogenetic resolution for many Carboniferous taxa makes it difficult to determine how the Hangenberg extinction and recovery intervals shaped actinopterygian diversity. Understanding the origin of later Carboniferous actinopterygian diversity requires a better understanding of earliest Carboniferous faunas and their interrelationships [16]. Explicit phylogenetic treatment of early Carboniferous actinopterygians is critical in determining whether later Carboniferous actinopterygians represent Devonian survivors or a post-Hangenberg diversification.

New actinopterygian fossils from the Tournaisian provide an opportunity to improve our understanding of earliest Carboniferous faunas and test whether other osteichthyan lineages follow the tetrapod pattern. Here, we describe a new genus of actinopterygian from a three-dimensionally preserved partial skull from Blue Beach, Nova Scotia, a locality in the Tournaisian Horton Group. This locality has previously yielded a diverse tetrapod fauna, as well as undescribed actinopterygian and sarcopterygian material [8,15,17]. Mirroring trends in the tetrapod fauna of this locality, this specimen demonstrates close affinity to taxa more typical of the Devonian, with clear implications for end-Devonian extinction survivorship among vertebrates.

## 2. Material and methods

### 2.1. Preparation and photography

The specimen, NSM016GF025.001, was prepared under a stereomicroscope using a pin vice and consolidated with dilute polyvinyl acetate.

NSM016GF025.001 was photographed using a Nikon D200 DSLR with a macrolens. We photographed the specimen under alcohol immersion to increase contrast between bone and matrix.

### 2.2. Micro-CT and three-dimensional imaging

We conducted µCT scanning of NSM016GF025.001 in the McCaig Institute for Bone and Joint Health, University of Calgary, Calgary, Alberta. The specimen was scanned on a Skyscan1173 at 80 kV and 100 mA with a voxel size of 46.8 µm and reconstructed as a tomographic stack using nRecon 1.6.6.0. The resulting images were cropped in ImageJ and were subsequently imported into Amira 5.4.0 for imaging.

### 2.3. Phylogenetic analysis

We added the new taxon and *Lambeia pectinata* to the character matrix of Giles *et al*. [18]. We removed *Tegeolepis clarki*, as it was acting as a wildcard taxon following Anderson [19], and *Dicksonosteus* from the outgroup. We also added a character for the trajectory of the lateral dorsal aortae, which we coded from the literature (electronic supplementary material, S1). Our new specimen was coded directly from the specimen and from µCT, whereas *L. pectinata* was coded from the recent description of Mickle [20]. The resulting matrix contains 93 taxa and 266 characters (electronic supplementary material, S2).

We assessed phylogenetic relationships of actinopterygians using an equally weighted parsimony analysis in PAUP * 4.0a [21]. Ancestral states were calculated using DELTRAN and dashes were treated as gaps. Most parsimonious trees were found using a heuristic search, with 500 random addition sequences, five trees held at each step, nchuck = 10 000, chuckscore = 1 and the tree bisection and reconnection strategy enabled. Trees were rooted in a non-osteichthyan outgroup of *Entelognathus*, *Acanthodes*, *Cladodoides* and *Ozarcus*.

## 3. Systematic palaeontology

*Osteichthyes* Huxley [22]

*Actinopterygii* Cope [23]

*Avonichthys manskyi* gen. et sp. nov.

### 3.1. Etymology

Genus name for the Avon River, where Blue Beach is located. Species epithet for Chris Mansky, in honour of his many years of collecting, preparing, exhibiting and publicly presenting the fossils of Blue Beach.

### 3.2. Material

NSM016GF025.001 (Nova Scotia Museum), comprising the posterior portion of the skull, collected in the intertidal debris in the cove below the lighthouse, one of the most fossiliferous areas, by J.S.A. in 2013. While this is the most likely source of the specimen, given the large daily tides in the basin (approximately 40 feet every 12 hours), it is possible that the specimen was transported into the area. If this were the case, then the fossil would have originated in the slightly older Blue Beach Member of the Horton Group, but this is still Tournasian, so there is no substantial impact on our results.

### 3.3. Locality

Hurd Creek Member of the Horton Bluff Formation, near Hantsport, Nova Scotia. NSM016GF025.001 was recovered from undifferentiated, eroded material on the western shore of the Avon River.

### 3.4. Diagnosis

Actinopterygian uniquely possessing: bifurcation of dorsal aorta enclosed in canal with widely divergent, long subparallel grooves for the lateral dorsal aortae medial and posterior to the articulation for the first infrapharyngobranchial; relatively anterior spiracular embankment of intertemporal; massively ossified, horizontal quadrate; groove for the facial branch of the hyomandibular nerve and foramen for the internal mandibular branch of the facial nerve relatively anteriorly situated. Shares with *Moythomasia*: relatively long intertemporal. Differs from *Moythomasia*: no serrations or barbs on vermiform ornamentation.

## 4. Results

### 4.1. Description

NSM016GF025.001 preserves the posterior portion of the right skull roof, including the right supratemporal, intertemporal, dermosphenotic, parietal and frontal; part of the right suspensorium, including the dermohyal, hyomandibula and the posterior-most part of the quadrate; the posterior-most portion of the interorbital septum; the base of the parasphenoid and the posterior otic-occipital. Elements are disarticulated such that ventral structures, including the base of the parasphenoid and the posterior otic-occipital, are displaced right-laterally, dorsally and anteriorly relative to more dorsally situated structures.

**Figure 1.**
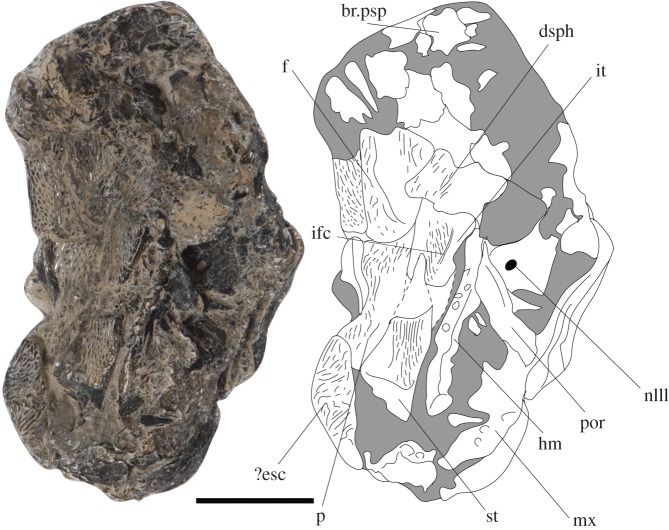
*Avonichthys manskyi* gen. et sp. nov. Photograph and interpretive drawing of specimen in dorsal view. Scale bar = 10 mm. br.psp, broken dorsal surface of parasphenoid; dsph, dermosphenotic; ?esc, indeterminate extrascapular; f, frontal; hm, hyomandibular; ifc, infraorbital canal; it, intertemporal; mx, maxilla; nIII, foramen for oculomotor nerve; p, parietal; por, post-orbital process; st, supratemporal.

**Figure 2.**
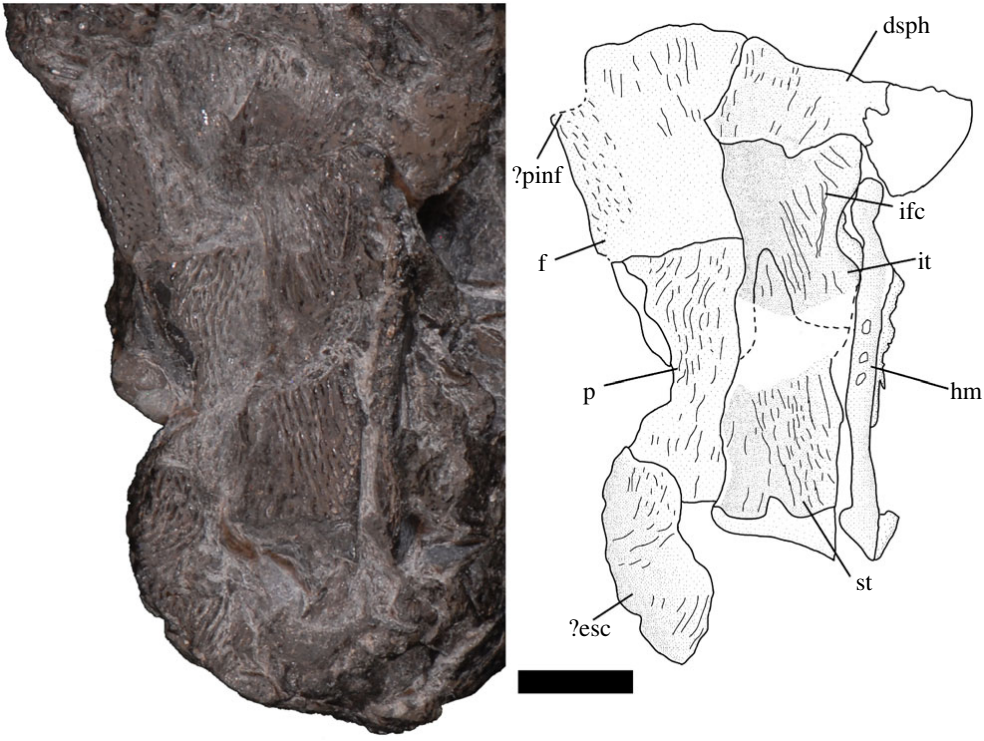
*Avonichthys manskyi* gen. et sp. nov. Photograph and interpretive drawing of dermal skull roof in dorsal view. Scale bar = 5 mm. dsph, dermosphenotic; ?esc, indeterminate extrascapular; f, frontal; hm, hyomandibular; ifc, infraorbital canal; it, intertemporal; p, parietal; ?pinf, pineal foramen; st, supratemporal.

Overall, the skull roof bones are rectangular and well ornamented with elevated ridges of glossy tissue that run subparallel to the long anteroposterior axis and form vermiform ornamentation typical of early actinopterygians (figures 1 and 2).

We interpret separate supratemporal and intertemporal ossifications, rather than a single dermopterotic ossification, based on a partial suture on the right side of the skull roof; it forms an acute ‘V’ with an anterior point (figures 1 and 2). The elements are damaged just posterior to this point, so the full extent of the suture is unknown. The supratemporal has a long anteroposterior axis, with its apex posterior to the suture between the frontal and the parietal. The posterior margin of this element shows an occipital flange devoid of ornamentation. Medially, the occipital flange is narrow along its anteroposterior axis, but flares out posteriorly towards its right lateral-most margin (figures 1 and 2). This flare is less pronounced than in taxa such as *Mimipiscis toombsi* and *Moythomasia durgaringa*. The relative length of the intertemporal is difficult to assess, as it is damaged and overlain by the displaced dermosphenotic anteriorly. Whereas the preserved extent is shorter than the supratemporal, it is long relative to the intertemporal in other actinopterygian genera (e.g. *Raynerius* and *Woodichthys*) and similar in relative length to the intertemporal of *Moythomasia* [24–26]. There is a shallow embayment on the lateral margin of the intertemporal that probably allowed the passage of the spiracular. The intertemporal is slightly eroded on its dorso-lateral extent, which has an exposed part of the infraorbital canal (figure 2). The intertemporal is similar in length to the supratemporal, but it is hard to determine the total extent as it is overlain by the displaced and rotated dermosphenotic. The dermosphenotic is damaged, leaving its posterior extent unknown; the anterior limb of this element extends below the matrix.

The right parietal is partially preserved. The displacement of the supratemporal and intertemporal dorsally and medially obscures the suture between those elements and the parietal (figures 1 and 2). It is overlain posteriorly by an indeterminate extrascapular, which marks the posterior end of the specimen. The parietal appears to be relatively long in comparison with the preserved extent of the frontal. The suture with the frontal is incompletely known, but appears to run anterolaterally. The frontal itself is poorly preserved, being obliterated laterally and anteriorly, and is overlain laterally by the intertemporal (figures 1 and 2). Much of the medial edge of the frontal is preserved—the right side of the midline suture. This appears straight and without significant interdigitation. The medial margin of the frontal is laterally indented anteriorly. This may be the right-posterior corner of the pineal foramen.

**Figure 3.**
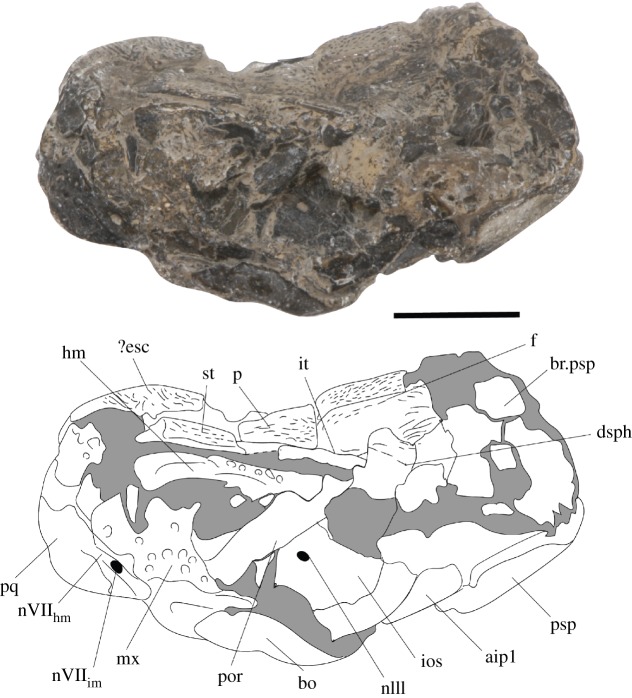
*Avonichthys manskyi* gen. et sp. nov. Photograph and interpretive drawing of specimen in lateral view. Scale bar = 10 mm. aip1, articulation for the first infrapharygobranchial; bo, basioccipital; br.psp, broken dorsal surface of parasphenoid; dsph, dermosphenotic; ?esc, indeterminate extrascapular; f, frontal; hm, hyomandibular; ios, interorbital septum; it, intertemporal; mx, maxilla; nIII, foramen for oculomotor nerve; nVII_hm_, groove for the hyomandibular branch of facial nerve; nVII_im_, foramen for the internal mandibular branch of the facial nerve; p, parietal; por, post-orbital process; pq, palatoquadrate; psp, parasphenoid; st, supratemporal.

The right suspensorium is displaced such that the long axis of the fused dermohyal and hyomandibula lies parallel to the skull roof (figures 2 and 3). The dermohyal displays prominent dermal texturing. The hyomandibula is displaced posteriorly from its point of articulation with the skull roof and post-orbital process. Ventrally, the posterior-most portion of the quadrate is preserved—it is large and robust and is tightly sutured to a densely denticulate quadrate ramus of the pterygoid. The groove for the facial branch of the hyomandibular nerve runs obliquely along the lateral surface of the quadrate and is pierced at the midpoint by a foramen serving the internal mandibular branch of the facial nerve (figure 3). The arrangement of this groove and foramen is similar to the condition described in *Mimipiscis* by Gardiner [27], but is more horizontal in orientation.

The post-orbital process descends from the dorsal braincase anterior to the suspensorium. The ventral extent of this element cannot be discerned, as it reaches the plane of shear. The posterior-most portion of the interorbital septum is displaced and rotated such that its ventral extent is lateral to its dorsal extent. The surface of the interorbital septum preserves a foramen—this probably served the oculomotor nerve (figure 3).

**Figure 4.**
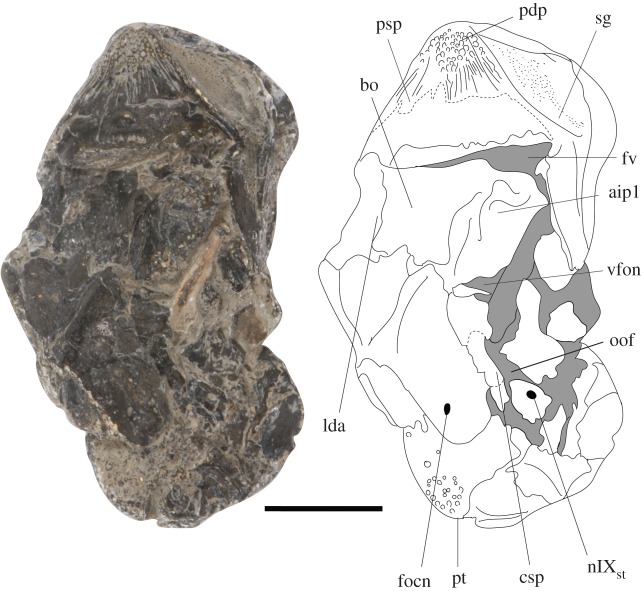
*Avonichthys manskyi* gen. et sp. nov. Photograph and interpretive drawing of specimen in ventral view. Scale bar = 10 mm. aip1, articulation for the first infrapharygobranchial; bo, basioccipital; csp, craniospinal process; focn, foramen for the occipital nerve; fv, ventral fissure; lda, lateral dorsal aorta; nIX_st_, foramen for the supratemporal branch of glossopharyngeal nerve; oof, otic-occipital fissure; pdp, parasphenoid denticle plate; psp, parasphenoid; pt, pterygoid; sg, spiracular groove; vfon, vestibular fontanelle.

**Figure 5.**
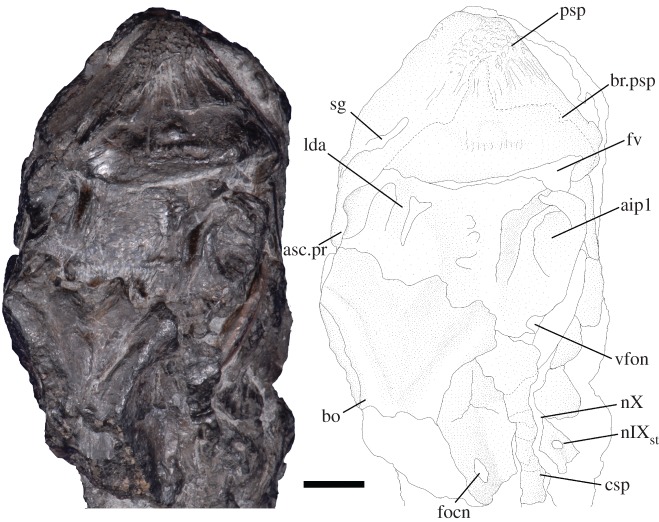
*Avonichthys manskyi* gen. et sp. nov. Photograph and interpretive line drawing of otic-occipital region in ventral view. Scale bar = 5 mm. aip1, articulation for the first infrapharygobranchial; asc.pr, ascending process of the parasphenoid; bo, basioccipital; csp, craniospinal process; focn, foramen for the occipital nerve; fv, ventral fissure; lda, lateral dorsal aorta; nIX_st_, foramen for the supratemporal branch of glossopharyngeal nerve; nX, widening for the passage of the vagus nerve; psp, parasphenoid; sg, spiracular groove; vfon, vestibular fontanelle.

**Figure 6.**
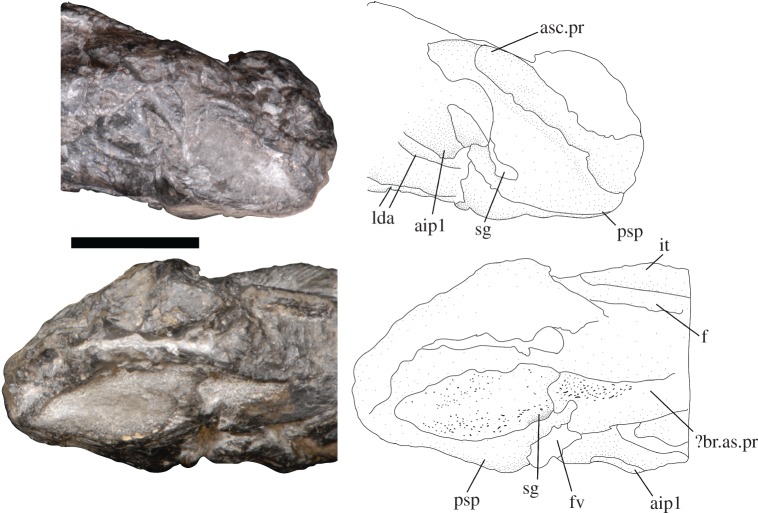
*Avonichthys manskyi* gen. et sp. nov. Photograph and interpretive line drawing of the parasphenoid in right (top) and left (bottom) lateral view. Scale bar = 10 mm. aip1, articulation for the first infrapharygobranchial; asc.pr, ascending process of the parasphenoid; ?br.asc.pr, broken component of the ascending process; f, frontal; fv, ventral fissure; it, intertemporal; lda, lateral dorsal aorta; psp, parasphenoid; sg, spiracular groove.

**Figure 7.**
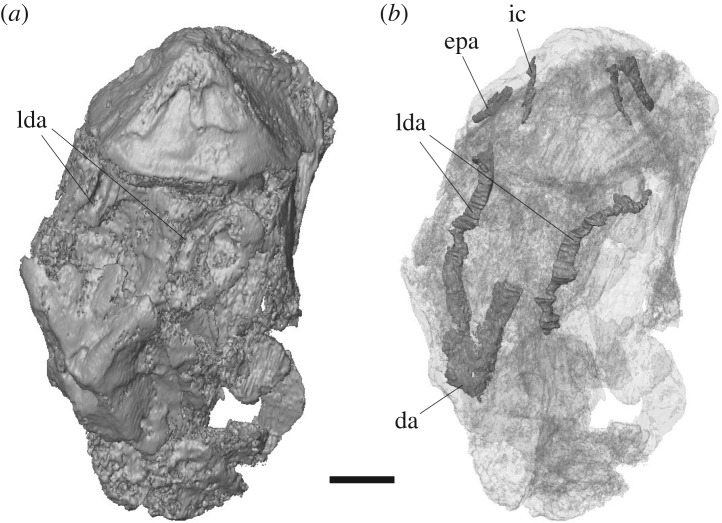
Internal structure of the braincase of *Avonichthys manskyi* gen. et sp. nov. rendered from µCT. Scale bar = 5 mm. (*a*) Volume rendering of specimen in ventral view. (*b*) Transparent µCT rendering of specimen in ventral view with internal canals segmented. da, dorsal aorta; epa, efferent pseudobranchial artery; ic, internal carotid; lda, lateral dorsal aorta.

The braincase is missing anterior to the apex of the base of the parasphenoid—the anterior-most preserved surface of the parasphenoid preserves an indentation which may represent part of the buccohypophyseal canal, but the complete canal is not preserved. The posterior plate of the paraphenoid is broad, extensive and anteriorly restricted (figures 4 and 5). The posterior palatal surface has broken, so the extent of denticulation cannot be fully determined, but circular denticle bases are identifiable on the anterior-most portion of the ventral surface. The erosion of the parasphenoid has exposed the dorsal margin of the parasphenoid in ventral view, permitting a determination of its full posterior extent. There is an indentation in this margin for the attachment of the subcephalic muscles. No parotic toothplates are preserved in this specimen, and the damage renders it impossible to evaluate if there is a point of articulation for these elements (figures 4 and 5). The spiracular groove is preserved on the lateral surface of the base of the parasphenoid (figure 6); it is shallow and positioned posterodorsally. The spiracular groove appears to be bounded dorsally by a strut on the anterior ascending process of the parasphenoid, although it is weathered, so the full morphology of the strut remains unknown. The ascending process is reduced in extent in comparison to the ascending process of *Howqualepis* [28,29], but does reach the level of the ventral otic fissure (figure 6). Vascular canals through the basisphenoid–parasphenoid complex can be seen using µCT (figure 7). The internal carotids enter the parasphenoid posteriorly, within the ventral cranial fissure, and begin to converge anteriorly. A second pair of canals are visible in µCT and run medially and anteriorly towards the internal carotids; these probably served the pseudobranchials (figure 7). There is no foramen for these canals in the preserved extent of the parasphenoid.

Part of the posterior otic-occipital is preserved. The specimen is broken just anterior to the posterior end of the braincase, producing an oblique transverse section of the canals for the dorsal aorta and notochord, and the foramen magnum. The foramen for the occipital nerve is preserved on the right lateral otic-occipital, posterior to the confluence of the grooves of the lateral dorsa aortae into the aortic canal (figures 4 and 5). This foramen is overlain by the craniospinal process. The craniospinal process forms the posteroventral margin of the otoccipital fissure, which runs posterodorsally. The otic-occipital fissure widens for the passage of the vagus nerve. There is another foramen dorsal to this fissure, probably serving the glossopharyngeal nerve. The otic-occipital fissure appears to be continuous with the vestibular fontanelle, but the shape and extent of the vestibular fontanelle is obscured by the overall crushed nature of the otic region (figures 4 and 5).

The ventral surface of the otic-occipital is conspicuously marked by paired, open grooves for the passage of the lateral dorsal aortae. These grooves run parallel for approximately half the length of the otoccipital region, at which point the lateral dorsal aortae became fully enclosed by the basioccipital (figures 4 and 5). Three-dimensional visualization of the canals serving the lateral dorsal aortae shows that the confluence of the lateral dorsal aortae into a common dorsal aorta (figure 7) occurs within the bone of the otic-occipital. A pair of oblong facets lateral to the lateral dorsal aortae are interpreted as the articulation facets for the first infrapharyngobranchials.

### 4.2. Phylogenetic analysis

**Figure 8.**
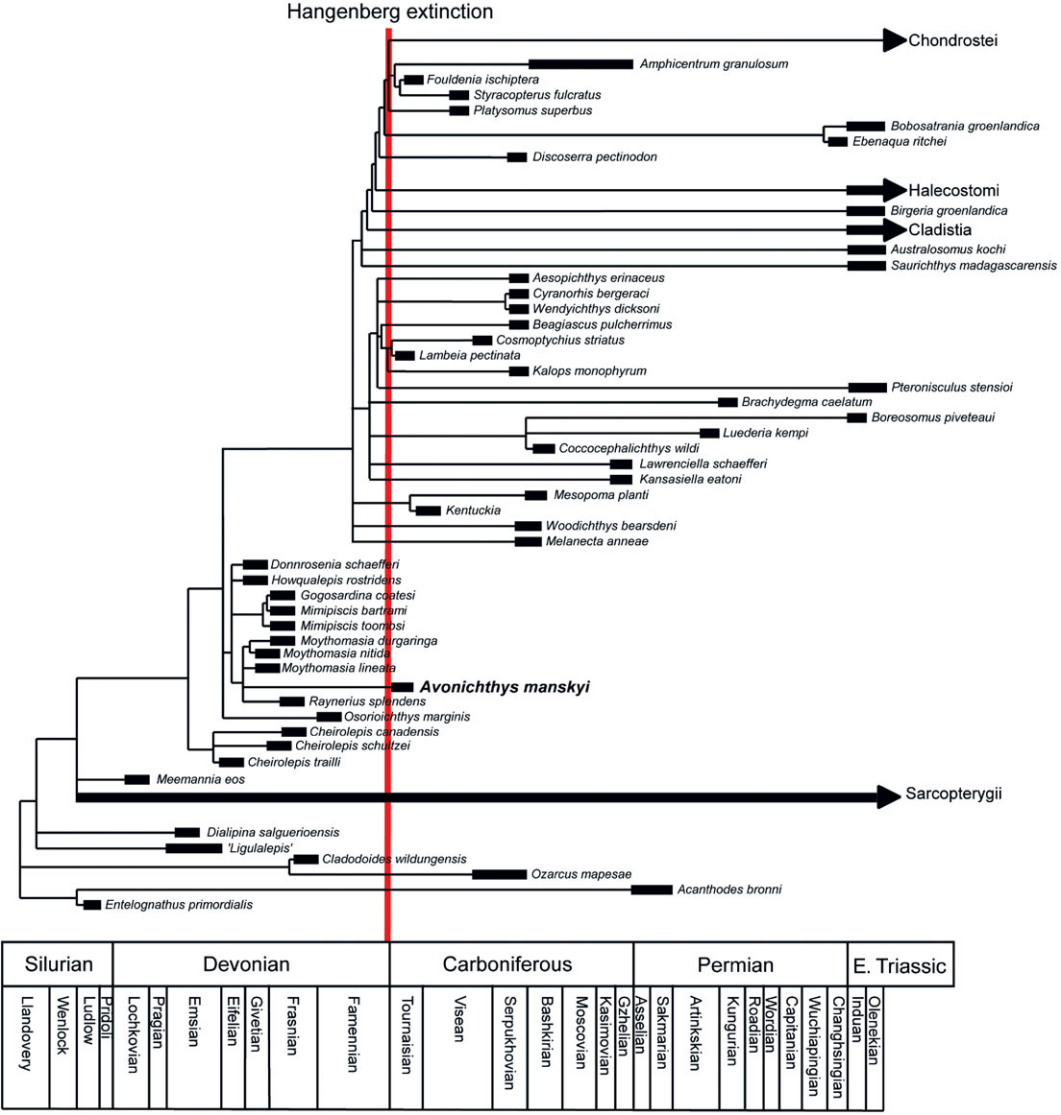
Phylogenetic and temporal placement of *Avonichthys manskyi* gen. et sp. nov. Adams consensus of 138 064 trees. Tree length = 1325 steps, CI = 0.223 and RI = 0.641. Taxon ages derived from the literature (electronic supplementary material, S4).

The analysis of osteichthyan relationships recovered 138 064 most parsimonious trees, with a tree length of 1325 steps. The consistency index was 0.223, and the retention index was 0.641. In the Adams consensus, *Donnrosenia*, *Howqualepis*, the clade containing mimiids and the clade containing moythomasiids are recovered in a polytomy (figure 8). This clade is supported by apomorphies in c.25, pineal foramen present; c.43, one pair of extrascapulars; c.110, an operculum at least twice as high as suboperculum; c.112, anterodorsal process of suboperculum present; c.174, parasphenoid with multifid anterior margin; c.193, ampulla of posterior semicircular canal separated from cranial cavity by a short length of canal and c.227, presupracleithrum present (numbers refer to characters in electronic supplementary material, S2). *Avonichthys manskyi* is found within this latter clade, as a member of a polytomy including *Moythomasia* and *Raynerius* (figure 8). This clade is supported by apomorphies in c.12, sensory canal on premaxilla present; c.43, two pairs of extrascapulars; c.47, medially directed branch of sensory canal in extrascapulae absent; c.119, dorsal-most branchiostegal ray in series deeper than adjacent ray; c.156, grooves for lateral dorsal aorta broadly separated and subparallel in anterior half of otic region; c.206, scales with well-developed pores absent; c.248, many dorsal scales anterior to dorsal fin extending to the posterior of skull roof and c. 261, median neural spines present in the caudal region (numbers refer to characters in electronic supplementary material, S2) . In the majority rule consensus, the *Raynerius* is a sister taxon to a polytomy of [*M. durgaringa* + *Moythomasia lineata* + *M. durgaringa*] + *A. manskyi*] in 52% of parsimonious trees (electronic supplementary material, S3). As the taxa in this polytomy are known exclusively from the Late Devonian of Australia and Europe, this result represents a significant temporal and range expansion of this lineage.

## 5. Discussion

### 5.1. Phylogenetic relationships of earliest Mississippian fishes from Eastern Canada

Phylogenetic analysis of *A. manskyi* shows the presence of an early diverging actinopterygian taxon in the earliest Tournaisian of Atlantic Canada (figure 8). Specifically, we recover *A. manskyi* as part of a polytomy including moythomasiids and *Raynerius*. This clade is, in turn, the sister group to mimiids. This represents a further indication of widespread survivorship of Devonian actinopterygian lineages into the Mississippian [26]. The position of *A. manskyi* is supported by the dorsal aortae open well posterior to the articulation for the first infrapharyngobranchial, the large open spiracular groove, a triangular anteriorly restricted parasphenoid that does not cross the ventral cranial fissure and the well-developed persistent ventral cranial fissure. This phylogenetic placement makes *A. manskyi* the earliest diverging actinopterygian lineage currently known to have survived the Hangenberg extinction. What is less clear is the phylogenetic relationship between *A. manskyi* and the broader diversity of Carboniferous actinopterygians from the Tournaisian and later. Several other actinopterygians are known from isolated remains from Blue Beach, including a second, larger, actinopterygian neurocranium (BWC 536), a partial skeleton of a deep-bodied form (BWC 1029) possibly attributable to the Platysomoidea and a mandible from a very large actinopterygian (CM 9827), as well as scales and isolated remains possibly attributable to several Palaeozoic actinopterygian ‘wastebasket’ taxa, including *Paleoniscum*, *Rhadinichthys* and *Acrolepis* [15]. A more extensive actinopterygian fauna is present from another Horton Group locality in New Brunswick [20], with an overall similar list of taxa: one or several species attributed to *Rhadinichthys*, a species probably incorrectly attributed to *Palaeoniscum*, a species attributed to *Elonichthys*, a possible *Canobius* and the possible elonichthyid *L. pectinata* [20]. Although we have not explicitly treated these taxa in our analysis here, rhadinichthyids (*Wendyichthys*) and elonichthyids (*Mansfieldiscus*) are both deeply nested within a more derived radiation of non-neopterygian actinopterygians, suggesting that much of the Palaeozoic actinopterygian radiation may have occurred by the earliest Carboniferous.

### 5.2. Actinopterygian survivorship across the Hangenberg extinction

The Devonian–Carboniferous transition is marked by at least two marine mass extinction events, the Kellwasser event at the Frasnian–Famennian boundary and the Hangenberg event at the Famennian–Tournaisian boundary. Of these two, the Kellwasser has a greater impact on marine invertebrate diversity [2,13]. However, the vertebrate communities of the Upper Devonian differ substantially from those of the Lower Carboniferous, and it has been argued that the Hangenberg represents a major extinction of vertebrate lineages, even if the impact on invertebrates is more moderate [13]. The presence of a classic Devonian actinopterygian lineage in the Horton Bluff Formation hints that post-Hangenberg recovery fauna may retain a more phylogenetically inclusive sample of Famennian actinopterygian diversity than previously appreciated. The only Devonian actinopterygian lineages which appear to go extinct before the Carboniferous are cheirolepids, which do not appear after the Frasnian [30], howqualepids, restricted to the Givetian [31], and basal-most forms such as *Meemannia*, *Ligulalepis* and *Dialipina*, which are restricted to the earliest Devonian [32–34]. The relative rarity of articulated actinopterygian fossils makes it difficult to determine whether these lineages reached the end of the Devonian, or whether they went extinct prior to the Hangenberg. One possibility is that the replacement of ‘archaic’ Devonian-type actinopterygian lineages by ‘advanced’ Carboniferous-type clades in the aftermath of the Hangenberg event was a function of differential diversification in the aftermath of the extinction rather than a function of clade selection within the mass extinction itself. Future approaches to the impact of the end-Devonian mass extinction on actinopterygian diversity should distinguish between the extent and selectivity of the mass extinction itself versus diversification patterns within the Lower Carboniferous recovery fauna.

This secondary question, whether actinopterygians underwent an adaptive radiation in the earliest Carboniferous, is more difficult to assess, as the phylogenetic relationships of the vast majority of Carboniferous actinopterygians remain unresolved. Although it is certainly the case that actinopterygian communities achieved high diversity within the Mississippian (e.g. [24,35,36]), it is unclear whether this diversity appeared suddenly or whether it accrued gradually over the Late Devonian and Early Carboniferous. Further phylogenetic work incorporating broader character samples (e.g. [37]) and more fully sampling Carboniferous and Permian actinopterygian diversity is necessary [18].

More inclusive survivorship of actinopterygians across the Hangenberg opens up the possibility that actinopterygian diversity may more closely align with the emerging pattern of mass survivorship among tetrapods [8,9] and dipnoans [10,12]. An extinction of archaic tetrapods has been previously proposed based on the apparent disappearance of ichthyostegalid-grade tetrapods [6], but recent work has shown that Devonian ichthyostegalid-grade tetrapods persist into the Tournaisian [8,9] and later [38], and that Carboniferous-type whatcheeriid-grade tetrapods are present in Devonian assemblages [39,40]. Similarly, recent work on dipnoans also shows a high diversity of this group in the Tournaisian [10], and several recent studies [11,12] have shown that many major post-Devonian lungfish lineages may have diverged as early as the Frasnian, well before the Hangenberg extinction. This suggests that the vertebrate signal of the Hangenberg extinction may be more complicated than previously thought, with extinction either concentrated within certain taxa (e.g. ‘placoderms’), within certain environments (marine versus freshwater selectivity) or in more subtle rearrangements of community structure without a clear extinction signal. Although the history of low collection effort in the Tournaisian makes it difficult to distinguish between these possibilities, increased collection in Early Tournaisian deposits, such as at Blue Beach, is a step towards a better understanding of this transitional period.

## Supplementary Material

Supplementary element 1

## Supplementary Material

Supplementary element 2

## Supplementary Material

Supplementary element 3

## Supplementary Material

Supplementary element 4

## Supplementary Material

Supplementary element 5

## Supplementary Material

Supplementary element 6

## Supplementary Material

Supplementary element 7

## Supplementary Material

Supplementary element 8

## Supplementary Material

Supplementary element 9

## Data accessibility

Our data are available as electronic supplementary material.
