# Supplementary material for: A primitive actinopterygian braincase from the Tournaisian of Nova Scotia
Source: R Soc Open Sci. 2018 May 16;5(5):171727. doi: 10.1098/rsos.171727 (PMC5990821; doi:10.1098/rsos.171727)
Supplement: Supplementary element 1 [file rsos171727supp1.docx]

This character describes the condition of the lateral dorsal aortae in taxa where these are present in grooves and diverge posterior to the ventral otic fissure. (1) represents the condition where these are broadly separated and subparallel in the anterior half of the ventral otic region. (2) represents the condition where these are not broadly separated and are angled towards convergence along most of their trajectory.

| Acanthodes bronni | Friedman and Brazeau 2010 |
| --- | --- |
| Acipenser brevirostrum |  |
| Aesopichthys erinaceus |  |
| Amia calva |  |
| Amphicentrum granulosum | Dyne 1939 |
| Atractosteus spatula |  |
| Australosomus kochi | Nielsen 1949 |
| Beagiascus pulcherrimus |  |
| Beishanichthys brevicaudalis |  |
| Birgeria groenlandica |  |
| Bobosatrania groenlandica |  |
| Boreosomus piveteaui | Nielsen 1942 |
| Brachydegma caelatum |  |
| Caturus furcatus |  |
| Cheirolepis canadensis |  |
| Cheirolepis schultzei |  |
| Cheirolepis trailli | Giles et al. 2015 |
| Chondrosteus acipenseroides |  |
| Cladodoides wildungensis | Maisey 2005 |
| Coccocephalichthys wildi | Poplin and Veran 1996 |
| Cosmoptychius striatus | Schaeffer 1971 |
| Cyranorhis bergeraci |  |
| Dapedium LIAS |  |
| Dapedium pholidotum |  |
| Dialipina salguerioensis |  |
| Diplocercides kayseri |  |
| Dipteronotus ornatus |  |
| Discoserra pectinodon |  |
| Donnrosenia schaefferi |  |
| Dorsetichthys bechei |  |
| Ebenaqua ritchei |  |
| Elops hawaiensis |  |
| Entelognathus primordialis | Zhu et al. 2013 |
| Erpetoichthys calabaricus |  |
| Eusthenopteron foordi |  |
| Evenkia eunoptera |  |
| Fouldenia ischiptera |  |
| Fukangichthys longidorsalis |  |
| Glyptolepis groenlandica |  |
| Gogonasus andrewsae |  |
| Gogosardina coatesi |  |
| Guiyu oneiros |  |
| Hiodon alosoides |  |
| Howqualepis rostridens | Giles et al. 2015 |
| Hulettia americana | Schaeffer and Patterson 1984 |
| Ichthyokentema purbeckensis | Griffith and Patterson 1963 |
| Kalops monophyrum |  |
| Kansasiella eatoni | Watson 1928 |
| Kentuckia deani | Rayner 1951 |
| Lawrenciella schaefferi | Poplin 1984 |
| Lepisosteus osseus |  |
| Leptolepis bronni | Rayner 1937 |
| '''Ligulalepis''' |  |
| Luederia kempi | Schaeffer and Dalquest 1978 |
| Luganoia lepidosteoides |  |
| Macrepistius arenatus |  |
| Macrosemimimus lennieri |  |
| Macrosemius rostratus |  |
| Meemannia eos |  |
| Melanecta anneae |  |
| Mesopoma planti |  |
| Miguashaia bureaui |  |
| Mimipiscis bartrami | Choo 2011 – visible in Figure 6a |
| Mimipiscis toombsi | Gardiner 1984 |
| Moythomasia durgaringa | Long and Trijnastic 2010 – visible in Figure 5b |
| Moythomasia lineata |  |
| Moythomasia nitida |  |
| Obaichthys decoratus |  |
| Onychodus jandemarrai |  |
| Osorioichthys marginis |  |
| Osteolepis macrolepidotus |  |
| Ozarcus mapesae | Pradel et al. 2014 |
| Peltopleurus lissocephalus |  |
| Platysomus superbus |  |
| Polypterus bichir |  |
| Porolepis sp. |  |
| Propterus elongatus |  |
| Psarolepis romeri |  |
| Pteronisculus stensioi | Nielsen 1942 |
| Raynerius splendens | Giles et al. 2015 |
| Saurichthys madagascarensis |  |
| Scanilepis dubia |  |
| Semionotus elegans |  |
| Styloichthys changae | Friedman 2007 |
| Styracopterus fulcratus |  |
| Tanaocrossus kalliokoskii |  |
| Tetragonolepis semicincta |  |
| Venusichthys comptus |  |
| Watsonulus eugnathoides | Olsen 1984 |
| Wendyichthys dicksoni |  |
| Woodichthys bearsdeni | Coates 1998 |
| Moythomasia manskyi |  |
| Lambeia pectinata |  |

Choo B. 2011 Revision of the actinopterygian genus Mimipiscis (=Mimia) from the Upper Devonian Gogo Formation of Western Australia and the interrelationships of the early Actinopterygii. *Earth Environ. Sci. Trans. R. Soc. Edinburgh* **102**, 77–104. (doi:10.1017/S1755691011011029)

Dyne MB. 1939 The Skull of Amphicentrum Granulosum. *Proc. Zool. Soc. London* **109 B**, 195–210. (doi:10.1111/j.1096-3642.1939.tb00713.x)

Friedman M. 2007 Styloichthys as the oldest coelacanth: Implications for early osteichthyan interrelationships. *J. Syst. Palaeontol.* **5**, 289–343. (doi:10.1017/S1477201907002052)

Friedman M, Brazeau MD. 2010 A reappraisal of the origin and basal radiation of the Osteichthyes. *J. Vertebr. Paleontol.* **30**, 36–56. (doi:10.1080/02724630903409071)

Gardiner BG. 1984 The relationships of the palaeoniscid fishes, a review based on new specimens of Mimia and Moythomasia from the Upper Devonian of Western Australia. *Bull. Br. Museum Nat. Hist. Geol.* **37**, 173–428.

Giles S, Coates MI, Garwood RJ, Brazeau MD, Atwood R, Johanson Z, Friedman M. 2015 Endoskeletal structure in Cheirolepis (Osteichthyes, Actinopterygii), An early ray-finned fish. *Palaeontology* **58**, 849–870. (doi:10.1111/pala.12182)

Giles S, Darras L, Clément G, Blieck A, Friedman M. 2015 An exceptionally preserved Late Devonian actinopterygian provides a new model for primitive cranial anatomy in ray-finned fishes. *Proc. R. Soc. B Biol. Sci.* **282**, 20151485. (doi:10.1098/rspb.2015.1485)

John G, Patterson C. 1963 The structure and relationships of the Jurassic fish Ichthyokentema Purbeckensis. *Bull. Br. Museum Nat. Hist. Geol.* **8**, 1–378.

Long JA, Trinajstic K. 2010 The Late Devonian Gogo Formation Lägerstatte of Western Australia: Exceptional Early Vertebrate Preservation and Diversity. *Annu. Rev. Earth Planet. Sci.* **38**, 255–279. (doi:10.1146/annurev-earth-040809-152416)

Maisey JG. 2005 Braincase of the Upper Devonian Shark Cladodoides Wildungensis (Chondrichthyes, Elasmobranchii), With Observations on the Braincase in Early Chondrichthyans. *Bull. Am. Museum Nat. Hist.* **288**, 1–103. (doi:10.1206/0003-0090(2005)288<0001:BOTUDS>2.0.CO;2)

Nielsen E. 1949 *Studies on Triassic Fishes from East Greenland II. Australosomus and Birgeria*. Copenhagen, DK: C.A. Reitzels Forlag.

Nielsen E. 1942 *Studies on Triassic Fishes from East Greenland I. Glaucolepis and Boreosomus*. Copenhagen, DK: C.A. Reitzels.

Olsen PE. 1984 The skull and pectoral girdle of the parasemionotid fish Watsonulus eugnathoides from the Early Triassic Sakamena Group of Madagascar, with comments on the relationships of the holostean fishes. *J. Vertebr. Paleontol.* **4**, 481–499. (doi:10.1080/02724634.1984.10012024)

Poplin CM. 1984 Lawrenciella Schaefferi n.g., n.sp. (Pisces: Actinopterygii) and the use of endocranial characters in the classification of the Palaeonisciformes. *J. Vertebr. Paleontol.* **4**, 413–421. (doi:10.1080/02724634.1984.10012019)

Poplin CM, Véran M. 1996 A revision of the actinopterygian fish Coccocephalus wildi from the Upper Carboniferous of Lancashire. In *2. Studies on Carboniferous and Permian vertebrates - 1. Special Papers in Palaeontology*,

Pradel A, Maisey JG, Tafforeau P, Mapes RH, Mallatt J. 2014 A Palaeozoic shark with osteichthyan-like branchial arches. *Nature* **509**, 608–611. (doi:10.1038/nature13195)

Rayner DH. 1951 III.—On the Cranial Structure of an Early Palæoniscid, Kentuckia, gen. nov. *Trans. R. Soc. Edinburgh* **62**, 53–83. (doi:10.1017/S0080456800009248)

Rayner DH. 1937 II.—On Leptolepis bronni Agassiz. *J. Nat. Hist. Ser. 10* **19**, 46–74. (doi:10.1080/00222933708655239)

Schaeffer B. 1971 The Braincase of the Holostean Fish Macrepistius, with Comments on Neurocranial Ossification in the Actinopterygii. *Am. Muesum Novit.* **2459**, 1–34.

Schaeffer B, Dalquest WW. 1978 A palaeonisciform braincase from the Permian of Texas, with comments on cranial fissures and the posterior myodome. *AmMusNov*

Schaeffer B, Patterson C. 1984 Jurassic Fishes from the Western United States, With Comments on Jurassic Fish Distribution. *Am. Museum Novit.* , 1–86.

Watson DMS. 1928 On some Points in the Structure of Palaeoniscid and allied Fish. *Proc. Zool. Soc. London* **98**, 49–70.

Zhu M *et al.* 2013 A Silurian placoderm with osteichthyan-like marginal jaw bones. *Nature* **502**, 188–193. (doi:10.1038/nature12617)
