# Supplementary figures and images for: A primitive actinopterygian braincase from the Tournaisian of Nova Scotia
Source: R Soc Open Sci. 2018 May 16;5(5):171727. doi: 10.1098/rsos.171727 (PMC5990821; doi:10.1098/rsos.171727)

Majority-rule consensus tree

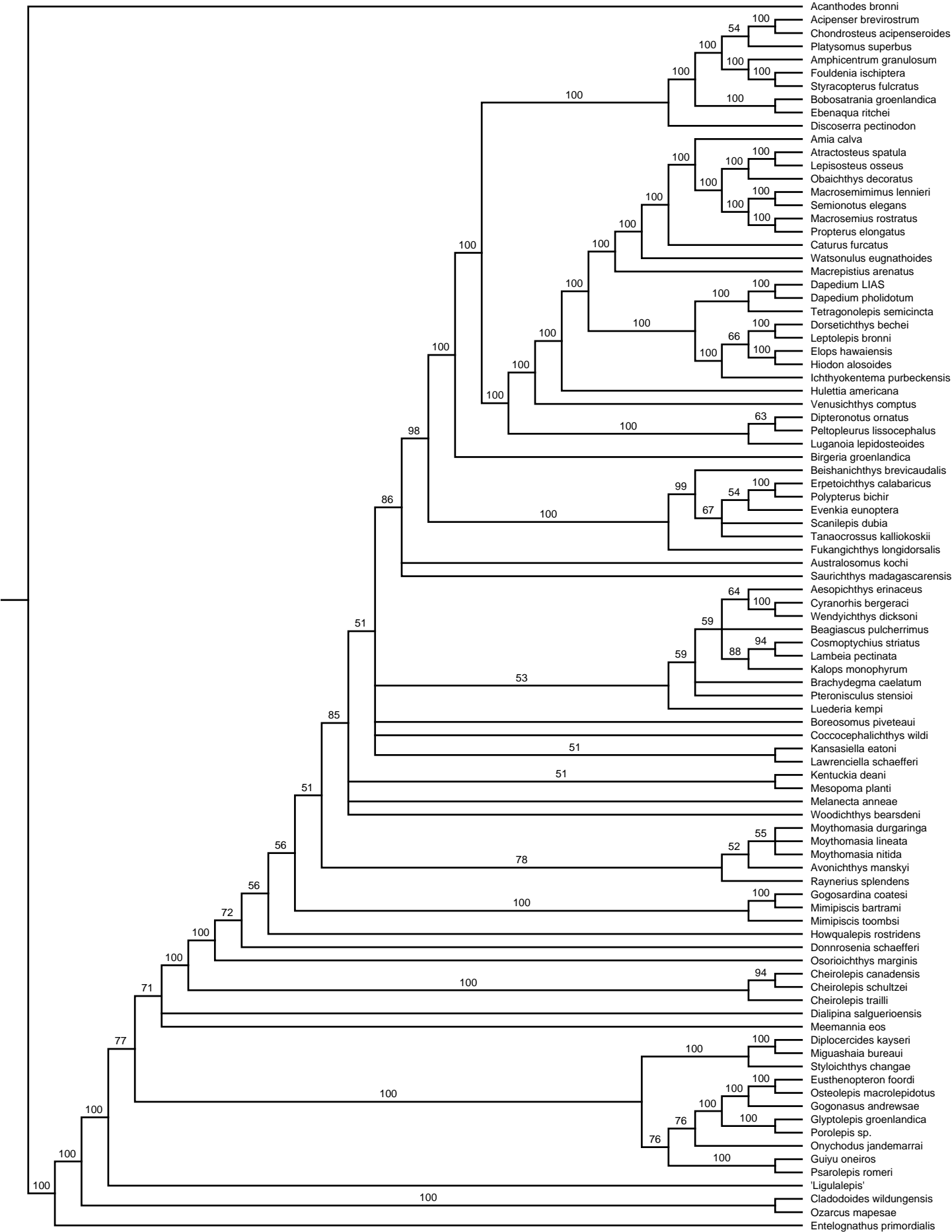

Supplement: Supplementary element 3 [file rsos171727supp3.pdf]

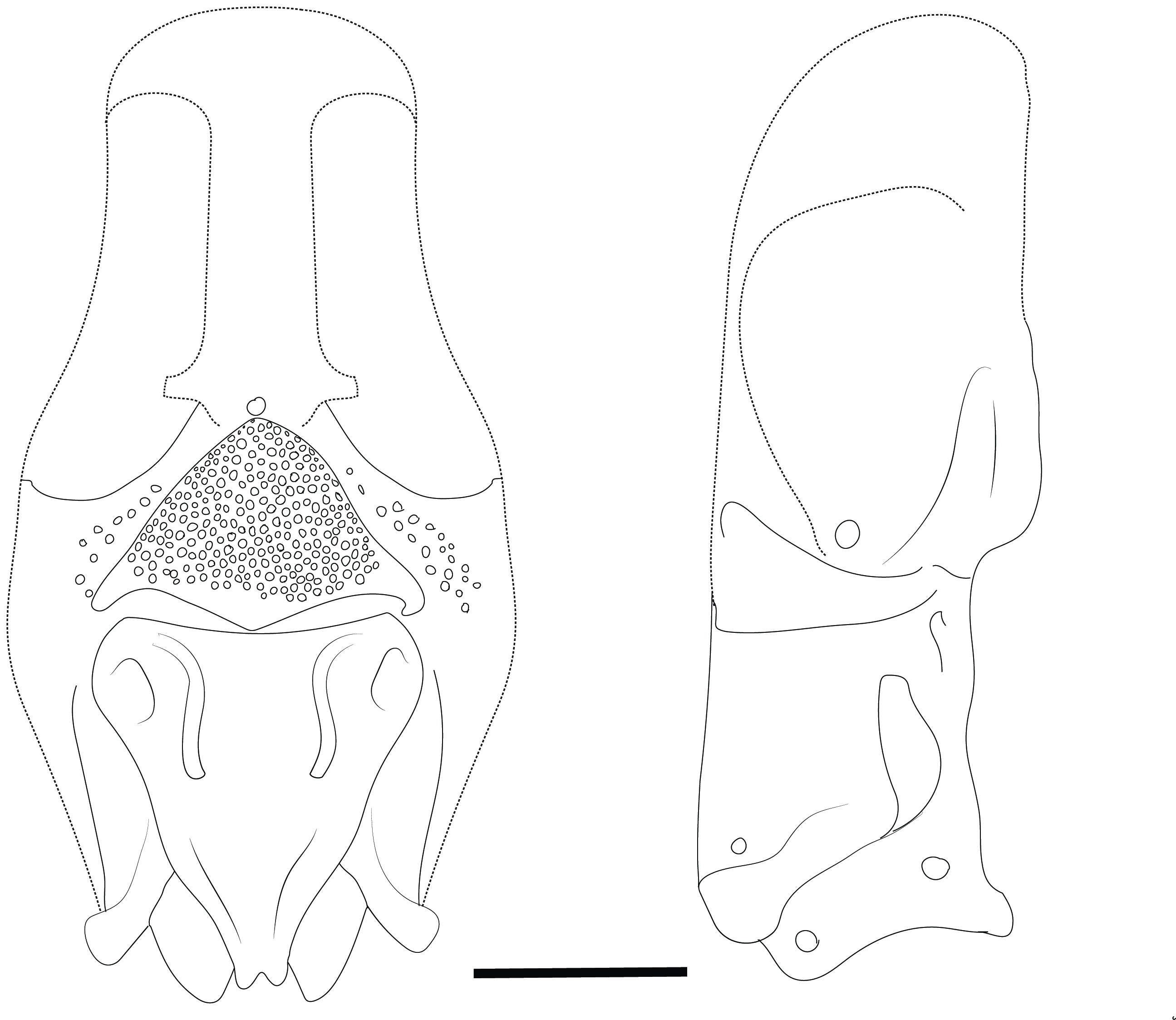

Supplement: Supplementary element 5 [file rsos171727supp5.jpg]

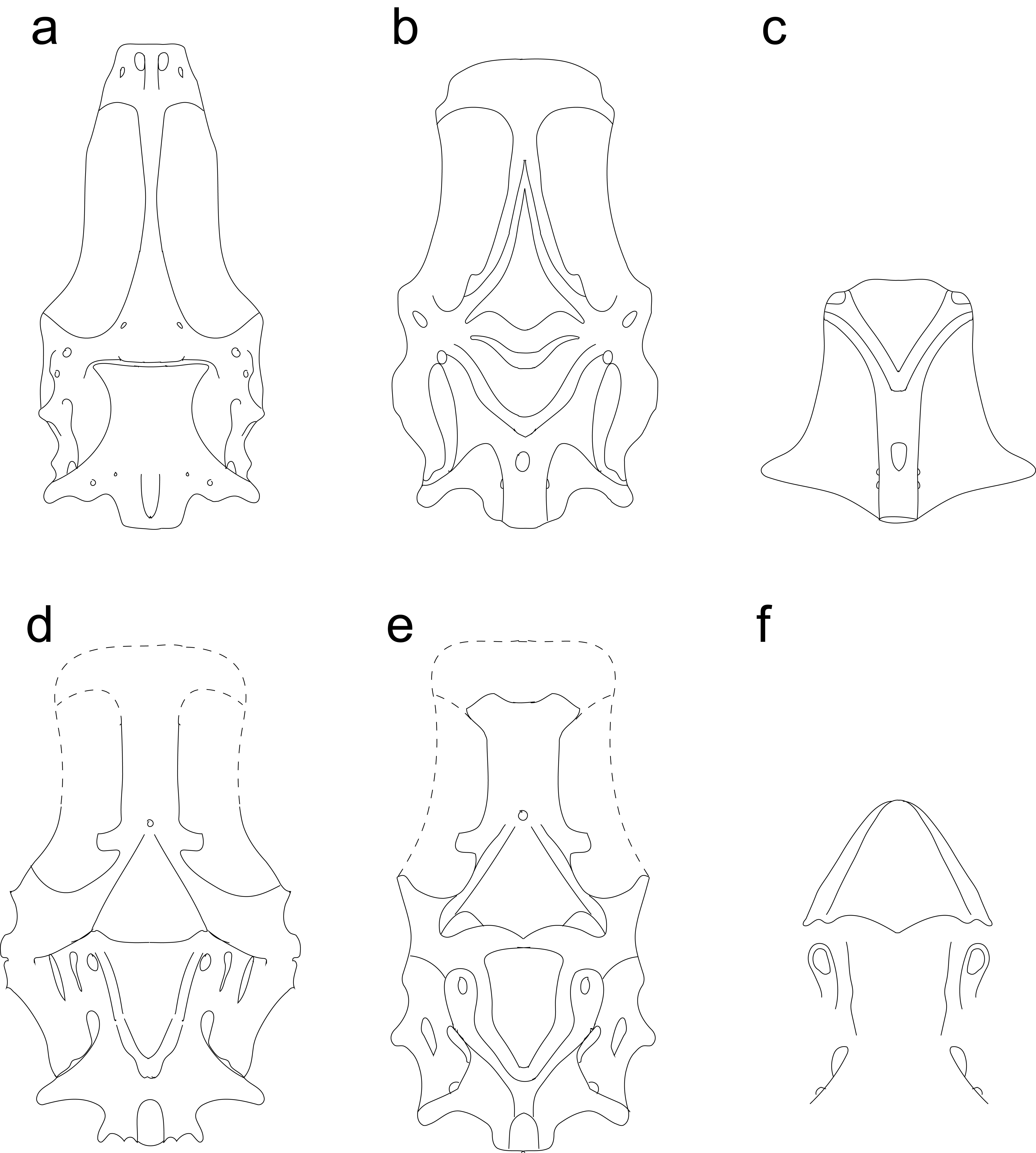

Supplement: Supplementary element 6 [file rsos171727supp6.jpg]

# Strict consensus tree

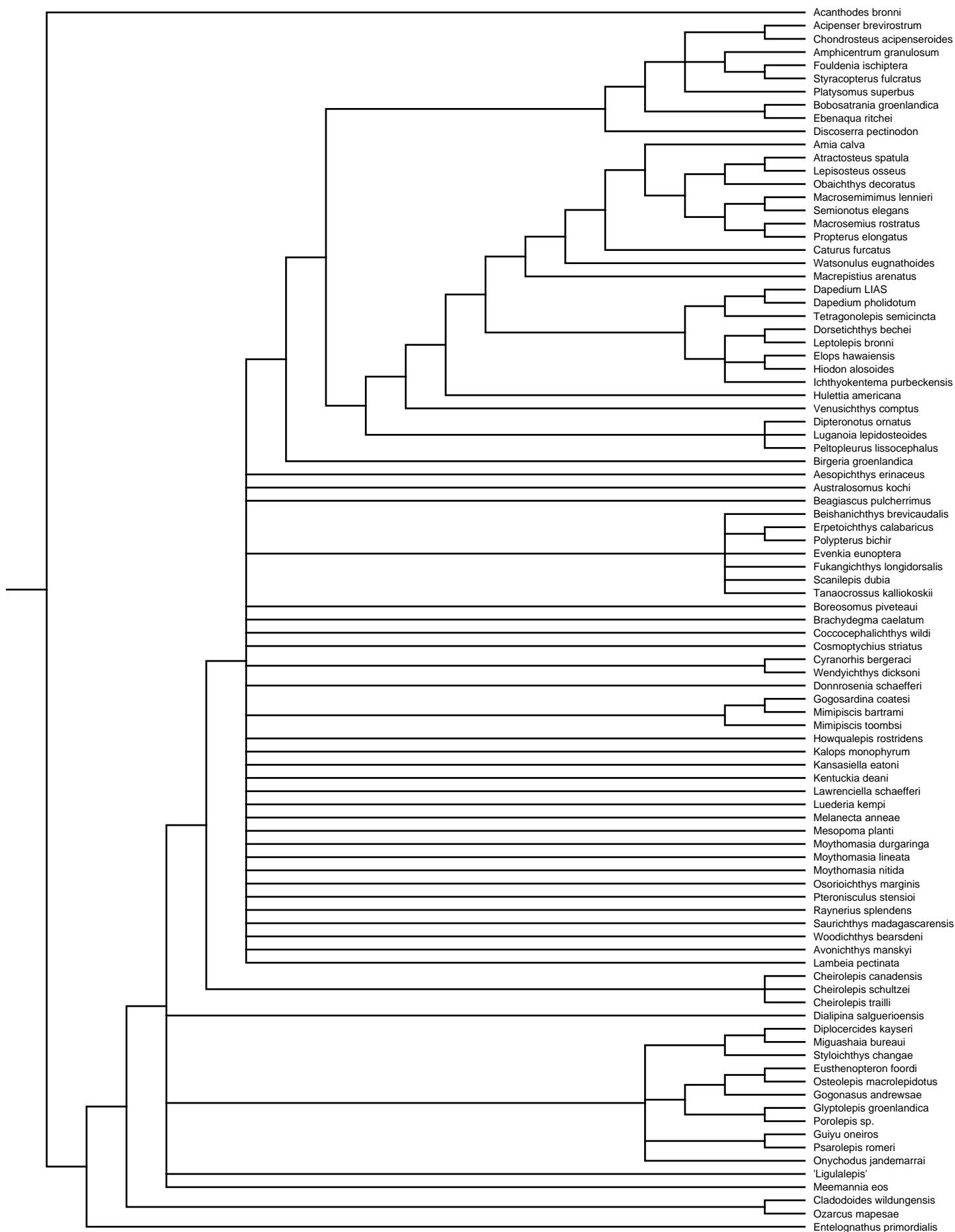

Supplement: Supplementary element 7 [file rsos171727supp7.pdf]
