# Supplementary material for: A primitive actinopterygian braincase from the Tournaisian of Nova Scotia
Source: R Soc Open Sci. 2018 May 16;5(5):171727. doi: 10.1098/rsos.171727 (PMC5990821; doi:10.1098/rsos.171727)
Supplement: Supplementary element 4 [file rsos171727supp4.docx]

| *Acanthodes bronni* | Early Permian (Asselian-Sakmarian) | Davis, Finarelli, and Coates 2012 |
| --- | --- | --- |
| *Acipenser brevirostrum* | Modern |  |
| *Aesopichthys erinaceous* | Early Serpukhovian (Bear Gulch) | Poplin and Lund 2000 |
| *Amphicentrum granulosum* | Upper Carboniferous | Dyne 1939 |
| *Australosomus kochi* | Early Triassic | Nielsen 1942 |
| *Beagiascus pulchermimus* | Early Serpukhovian (Bear Gulch) | Mickle, Lund, and Grogan 2009 |
| *Beishanichthys* | Early Triassic | Xu and Gao 2011 |
| *Birgeria groenlandica* | Early Triassic | Nielsen 1942 |
| *Bobastrania groenlandica* | Early Triassic | Stensio 1932 |
| *Boreosomus piveteaui* | Early Triassic (Griesbachian) | Nielsen 1942 |
| *Brachydegma caelatum* | Early Permian (Clear Fork) | Dunkle 1939 |
| *Cheirolepis canadensis* | Middle Frasnian (Miguasha) | Arratia and Cloutier 1996 |
| *Cheirolepis schultzei* | Devonian (Frasnian) | Arratia and Cloutier 2004 |
| *Cheirolepis trailli* | Devonian (late Eifelian) | Giles et al. 2015 |
| *Chondrosteus acipenseroides* | Early Jurassic (LIAS) | Egerton 1858 |
| *Cladodoides wildungensis* | Upper Frasnian | Maisey 2005 |
| *Coccocephalus wildi* | Upper Carboniferous | Poplin and Veran 1996 |
| *Cosmoptychius striatus* | Upper Visean | Watson 1928 |
| *Cyranorhis bergeraci* | Early Serpukhovian (Bear Gulch) | Lund and Poplin 1997 |
| *Dialipina salguerioensis* | Early Devonian | Schultze and Cumbaa 2001 |
| *Discoserra pectinodon* | Early Serpukhovian (Bear Gulch) | Lund 2000 |
| *Donnrosenia schaefferi* | Middle Devonian (Givetian) | Long et al. 2008 |
| *Ebenaqua ritchei* | Late Permian | Campbell and Le Duy Phuoc 1983 |
| *Entelognathus primordialis* | Silurian (Late Ludlow) | Zhu et al. 2013 |
| *Fouldenia ichiptera* | Tournaisian (348-347 Ma) | Sallan and Coates 2013 |
| *Gogosardina coatesi* | Lower Frasnian (Gogo) | Choo et al. 2009 |
| *Howqualepis rostridens* | Givetian (Mount Hewitt) | Choo 2009 |
| *Kalops monophyrum* | Early Serpukhovian (Bear Gulch) | Poplin and Lund 2000 |
| *Kansasiella eatoni* | Pennsylvanian (Bottom Virgilian) | Watson 1925, Poplin 1974 |
| *Kentuckia deani* | Late Tournaisian – early Visean | Rayner 1951; Giles and Friedman 2014 |
| *Lambeia pectinata* | Middle Tournaisian | Mickle 2017 |
| *Lawrenciella schaefferi* | Pennsylvanian (Bottom Virgilian) | Poplin 1984; Hamel and Poplin 2008 |
| *Ligulalepis* | Early Devonian (Emsian) | Basden and Young 2000 |
| *Luederia kempi* | Lower Permian (Lueders formation) | Schaeffer and Dalquest 1978 |
| *Meemannia eos* | Early Devonian (415 Ma) | Lu et al. 2016 |
| *Melanecta anneae* | Middle Carboniferous (Namurian) | Coates 1998 |
| *Mesopoma planti* | Early Pennsylvanian (Bashkirian) | Coates 1999 |
| *Mimipiscis bartrami* | Lower Frasnian (Gogo) | Choo 2011 |
| *Mimipiscis toombsi* | Lower Frasnian (Gogo) | Choo 2011 |
| *Moythomasia durgaringa* | Late Givetian-Early Frasnian | Choo 2015 |
| *Moythomasia lineata* | Late Givetian-Early Frasnian | Choo 2015 |
| *Moythomasia manskyi* | Earliest Tournaisian |  |
| *Moythomasia nitida* | Late Givetian-Early Frasnian | Choo 2015 |
| *Osorioichthys marginis* | Famennian | Taverne 1997 |
| *Ozarcus mapesae* | Upper Mississippian (Chesterian) | Pradel et al. 2014 |
| *Platysomus superbus* | Early Visean (Glencartholm) | Moy-Thomas and Bradley-Dyne 1938 |
| *Pteronisculus stensioi* | Triassic | Stensio 1921 |
| *Raynerius splendens* | Middle Frasnian | Giles et al. 2015 |
| *Saurichthys madagascarensis* | Early Triassic | Kogan et al. 2016 |
| *Styracopterus fulcratus* | Visean | Sallan and Coates 2013 |
| *Watsonulus eugnathoides* | Early Triassic | Olsen 1984 |
| *Wendyichthys dicksoni* | Early Serpukhovian (Bear Gulch) | Lund and Poplin 1997 |
| *Woodichthys bearsdeni* | Early Serpukhovian (Pendleian) | Coates 1998 |

Arratia G, Cloutier R. 1996 Reassessment of the morphology of *Cheirolepis canadensis* (Actinopterygii). In *Devonian Fishes and Plants of Miguasha, Quebec, Canada* (eds H-P Schultze, R Cloutier), pp. 165-198. Munchen, Germany: Verlag Dr. Friedrich Pfeil.

Arratia G, Cloutier R. 2004 A new cheirolepidid fish from the Middle-Upper Devonian of Red Hill, Nevada, USA. In *Recent Advances in the Origin and Early Radiation of Vertebrates* (eds G Arratia, MVH Wilson, R Cloutier), pp. 583-599. Munchen, Germany: Verlag Dr. Friedrich Pfeil.

Basden A, Young G, Coates M, Ritchie A. 2000 The most primitive osteichthyan braincase? *Nature* **403**, 185–188. (doi:10.1038/35003183)

Campbell KSW, Phuoc LD. 1983 A Late Permian actinopterygian fish from Australia. *Palaeontology*. **26(1)**, 33–70.

Choo B. 2015 A new species of the Devonian actinopterygian *Moythomasia* from Bergisch Gladbach, Germany, and fresh observations on *M. Durgaringa* from the Gogo Formation of Western Australia. *J. Vertebr. Paleontol.* **35**, e952817. (doi:10.1080/02724634.2015.952817)

Choo B. 2011 Revision of the actinopterygian genus Mimipiscis (=Mimia) from the Upper Devonian Gogo Formation of Western Australia and the interrelationships of the early Actinopterygii. *Earth Environ. Sci. Trans. R. Soc. Edinburgh* **102**, 77–104. (doi:10.1017/S1755691011011029)

Choo B. 2009 Basal Actinopterygian Fish from the Middle Devonian Bunga Beds of New South Wales, Australia. *Proc. Linn. Soc. New South Wales* **130**, 37–46.

Choo B, Long JA, Trinajstic K. 2009 A new genus and species of basal actinopterygian fish from the Upper Devonian Gogo Formation of Western Australia. *Acta Zool.* **90**, 194–210. (doi:10.1111/j.1463-6395.2008.00370.x)

Coates MI. 1999 Endocranial preservation of a Carboniferous actinopterygian from Lancashire, UK, and the interrelationships of primitive actinopterygians. *Philos. Trans. R. Soc. B Biol. Sci.* **354**, 435–462. (doi:10.1098/rstb.1999.0396)

Coates MI. 1998 Actinopterygians from the Namurian of Bearsden, Scotland, with comments on early actinopterygian neurocrania. *Zool. J. Linn. Soc.* **122**, 27–59. (doi:10.1006/zjls.1997.0113)

Davis SP, Finarelli JA, Coates MI. 2012 Acanthodes and shark-like conditions in the last common ancestor of modern gnathostomes. *Nature* **486**, 247–250. (doi:10.1038/nature11080)

Egerton P. 1858 On Chondrosteus, an Extinct Genus of the Sturionidaek, Found in the Lias Formation at Lyme Regis. *Philos. Trans. R. Soc. London* **148**, 871–885. (doi:10.1098/rstl.1858.0035)

Dunkle DH. 1939 A new paleoniscid fish from the Texas Permian. *Am. J. Sci.* **237**, 262–274.

Dyne MB. 1939 The Skull of Amphicentrum Granulosum. *Proc. Zool. Soc. London* **109 B**, 195–210. (doi:10.1111/j.1096-3642.1939.tb00713.x)

Giles S, Coates MI, Garwood RJ, Brazeau MD, Atwood R, Johanson Z, Friedman M. 2015 Endoskeletal structure in Cheirolepis (Osteichthyes, Actinopterygii), An early ray-finned fish. *Palaeontology* **58**, 849–870. (doi:10.1111/pala.12182)

Giles S, Darras L, Clément G, Blieck A, Friedman M. 2015 An exceptionally preserved Late Devonian actinopterygian provides a new model for primitive cranial anatomy in ray-finned fishes. *Proc. R. Soc. B Biol. Sci.* **282**, 20151485. (doi:10.1098/rspb.2015.1485)

Hamel M-H, Poplin C. 2008 the Braincase Anatomy of Lawrenciella Schaefferi, actinopterygian from the Upper Carboniferous of Kansas (USA). *J. Vertebr. Paleontol.* **28**, 989–1006. (doi:10.1671/0272-4634-28.4.989)

Kogan I, Romano C. 2016 Redescription of Saurichthys madagascariensis Piveteau, 1945 (Actinopterygii, Early Triassic), with implications for the early saurichthyid morphotype. *J. Vertebr. Paleontol.* **36**. (doi:10.1080/02724634.2016.1151886)

Long JA, Choo B, Young GC. 2008 A new basal actinopterygian fish from the Middle Devonian Aztec Siltstone of Antarctica. *Antarct. Sci.* **20**, 393–412. (doi:10.1017/S0954102008001144)

Lu J, Giles S, Friedman M, den Blaauwen JL, Zhu M. 2016 The Oldest Actinopterygian Highlights the Cryptic Early History of the Hyperdiverse Ray-Finned Fishes. *Curr. Biol.* **26**, 1602–1608. (doi:10.1016/j.cub.2016.04.045)

Lund R. 2000 The new Actinopterygian order Guildayichthyiformes from the Lower Carboniferous of Montana (USA). *Geodiversitas* **22(2)**, 171-206

Lund R, Poplin C. 1997 The Rhadinichthyids (Paleoniscoid actinopterygians) from the Bear Gulch limestone of Montana (USA, lower carboniferous). *J. Vertebr. Paleontol.* **17**, 466–486. (doi:10.1080/02724634.1997.10010996)

Lund R, Poplin C. 1997 The Rhadinichthyids (Paleoniscoid actinopterygians) from the Bear Gulch limestone of Montana (USA, lower carboniferous). *J. Vertebr. Paleontol.* **17**, 466–486. (doi:10.1080/02724634.1997.10010996)

Maisey JG. 2005 Braincase of the Upper Devonian Shark Cladodoides Wildungensis (Chondrichthyes, Elasmobranchii), With Observations on the Braincase in Early Chondrichthyans. *Bull. Am. Museum Nat. Hist.* **288**, 1–103. (doi:10.1206/0003-0090(2005)288<0001:BOTUDS>2.0.CO;2)

Mickle KE. 2017 The lower actinopterygian fauna from the Lower Carboniferous Albert shale formation of New Brunswick, Canada &amp;amp;ndash; a review of previously described taxa and a description of a new genus and species. *Foss. Rec.* **20**, 47–67. (doi:10.5194/fr-20-47-2017)

Mickle KE, Lund R, Grogan ED. 2009 Three new palaeoniscoid fishes from the Bear Gulch Limestone (Serpukhovian, Mississippian) of Montana (USA) and the relationships of lower actinopterygians. *Geodiversitas* **31**, 623–668. (doi:10.5252/g2009n3a6)

Moy-Thomas JA, Dyne MB. 1938 XVII. The Actinopterygian Fishes from the Lower Carboniferous of Glencartholm, Eskdale, Dumfriesshire. *Trans. R. Soc. Edinburgh* **59**, 437–480. (doi:10.1017/S0080456800009170)

Nielsen E. 1942 *Studies on Triassic Fishes from East Greenland I. Glaucolepis and Boreosomus*. Copenhagen, DK: C.A. Reitzels Forlag.

Nielsen E. 1949 *Studies on Triassic Fishes from East Greenland II. Australosomus and Birgeria*. Copenhagen, DK: C.A. Reitzels Forlag.

Olsen PE. 1984 The skull and pectoral girdle of the parasemionotid fish Watsonulus Eugnathoides from the Early Triassic Sakamena Group of Madagascar, with comments on the relationships of the holostean fishes. *J. Vertebr. Paleontol.* **4**, 481–499. (doi:10.1080/02724634.1984.10012024)

Poplin CM, Lund R. 2002 Two Carboniferous Fine-Eyed Palaeoniscoids (Pisces, Actinopterygii) From Bear Gulch (USA). *J. Paleontol.* **76**, 1014–1028. (doi:10.1666/0022-3360(2002)076<1014:TCFEPP>2.0.CO;2)

Poplin CM. 1984 Lawrenciella Schaefferi n.g., n.sp. (Pisces: Actinopterygii) and the use of endocranial characters in the classification of the Palaeonisciformes. *J. Vertebr. Paleontol.* **4**, 413–421. (doi:10.1080/02724634.1984.10012019)

Poplin CM, Véran M. 1996 A revision of the actinopterygian fish Coccocephalus wildi from the Upper Carboniferous of Lancashire. In *2. Studies on Carboniferous and Permian vertebrates - 1. Special Papers in Palaeontology*,

Poplin C, Lund R. 2000 Two new deep-bodied palaeoniscoid actinopterygians from Bear Gulch (Montana, USA, Lower Carboniferous). *J. Vertebr. Paleontol.* **20**, 428–449. (doi:10.1671/0272-4634(2000)020[0428:TNDBPA]2.0.CO;2)

Pradel A, Maisey JG, Tafforeau P, Mapes RH, Mallatt J. 2014 A Palaeozoic shark with osteichthyan-like branchial arches. *Nature* **509**, 608–611. (doi:10.1038/nature13195)

Rayner DH. 1951 On the Cranial Structure of an Early Palæoniscid, Kentuckia, gen. nov. *Trans. R. Soc. Edinburgh* **62**, 53–83. (doi:10.1017/S0080456800009248)

Sallan LC, Coates MI. 2013 Styracopterid (Actinopterygii) ontogeny and the multiple origins of post-hangenberg deep-bodied fishes. *Zool. J. Linn. Soc.* **169**, 156–199. (doi:10.1111/zoj.12054)

Schaeffer B, Dalquest WW. 1978 A palaeonisciform braincase from the Permian of Texas, with comments on cranial fissures and the posterior myodome. *AmMusNov*

Schultze H-P, Cumbaa SL. 2001 *Dialipina* and the characters of basal actinopterygians. In *Major Events in Early Vertebrate Evolution* (ed P Ahlberg), pp. 315-332. New York, NY: Taylor and Francis Inc.

Stensiö EA. 1932 Triassic fishes from East Greenland. *Meddel. Grønl* **83**: 17–117.

Taverne L. 1997 Osorioichthys marginis, ‘Paéonisciforme’ du Famennien de Belgique, et la phylogénie de Actinoptérygiens dévonians (Pisces). *Bull. L’institut R. des Sci. Nat. Belgique* **67**, 57–78.

Watson DMS. 1928 On some Points in the Structure of Palaeoniscid and allied Fish. *Proc. Zool. Soc. London* **98**, 49–70.

Watson DMS. 1925 The Structure of Certain Palaeoniscoids and the Relationships of that Group with other Bony Fish.

Xu GH, Gao KQ. 2011 A new scanilepiform from the Lower Triassic of northern Gansu Province, China, and phylogenetic relationships of non-teleostean Actinopterygii. *Zool. J. Linn. Soc.* **161**, 595–612. (doi:10.1111/j.1096-3642.2010.00645.x)

Zhu M *et al.* 2013 A Silurian placoderm with osteichthyan-like marginal jaw bones. *Nature* **502**, 188–193. (doi:10.1038/nature12617)
