# Supplementary material for: A primitive actinopterygian braincase from the Tournaisian of Nova Scotia
Source: R Soc Open Sci. 2018 May 16;5(5):171727. doi: 10.1098/rsos.171727 (PMC5990821; doi:10.1098/rsos.171727)
Supplement: Supplementary element 8 [file rsos171727supp8.docx]

Supplementary element 1. Character description and references for character added to phylogenetic data matrix.

Supplementary element 2. Data matrix used for phylogenetic analysis in nexus format. Also available on Dryad.

Supplementary element 3. Phylogenetic placement of *Avonichthys manskyi* gen. et sp. nov. Majority-rule consensus of 13,0864 most parsimonious trees. Tree length = 1325 steps, CI = 0.223, and RI = 0.641.

Supplementary element 4. Table of stratigraphic placements and references for taxa in figure 8.

Supplementary element 5. Line drawing reconstruction of *Avonichthys manskyi* gen. et sp. nov. in ventral (left) and left lateral (right) view. Scale bar = 5 mm.

Supplementary element 6. Comparative line drawings of actinopterygian taxa in ventral view. A., *Australosomus* spp. (Nielsen 1949); B., *Coccocephalichtys wildi* (Poplin and Veran 1996),;C., *Kentuckia deani* (Rayner 1951); D., *Raynerius splendens* (Giles et al. 2015); *E. Moythomasia durgaringa* (Gardiner 1984); F., *Avonichthys manskyi* gen. et sp. nov.

Supplementary element 7. Phylogenetic placement of *Avonichthys manskyi* gen. et sp. nov. Strict consensus of 6720 most parsimonious trees. Tree length = 1323 steps, CI = 0.224, and RI = 0.642.

Supplementary element 8. Captions for supplementary elements.

Supplementary element 9. Surface rendition of whole specimen in .wrl format. Available on Dryad
